# Senolytic Vaccines from the Central and Peripheral Tolerance Perspective

**DOI:** 10.3390/vaccines12121389

**Published:** 2024-12-10

**Authors:** Mariia I. Vasilieva, Rimma O. Shatalova, Kseniia S. Matveeva, Vadim V. Shindyapin, Ekaterina Minskaia, Roman A. Ivanov, Daniil V. Shevyrev

**Affiliations:** 1 Research Center for Translational Medicine, Sirius University of Science and Technology, Federal Territory Sirius, Krasnodarsky Krai, Sirius 354349, Russia; 2 Research Center for Genetics and Life Sciences, Sirius University of Science and Technology, Federal Territory Sirius, Krasnodarsky Krai, Sirius 354349, Russia; shindyapinvadim1996@yandex.ru

**Keywords:** ageing, senescence, senolytic vaccines, senescent-associated antigens, senescent-specific antigens, central tolerance, peripheral tolerance

## Abstract

Preventive medicine has proven its long-term effectiveness and economic feasibility. Over the last century, vaccination has saved more lives than any other medical technology. At present, preventative measures against most infectious diseases are successfully used worldwide; in addition, vaccination platforms against oncological and even autoimmune diseases are being actively developed. At the same time, the development of medicine led to an increase in both life expectancy and the proportion of age-associated diseases, which pose a heavy socio-economic burden. In this context, the development of vaccine-based approaches for the prevention or treatment of age-related diseases opens up broad prospects for extending the period of active longevity and has high economic potential. It is well known that the development of age-related diseases is associated with the accumulation of senescent cells in various organs and tissues. It has been demonstrated that the elimination of such cells leads to the restoration of functions, rejuvenation, and extension of the lives of experimental animals. However, the development of vaccines against senescent cells is complicated by their antigenic heterogeneity and the lack of a unique marker. In addition, senescent cells are the body’s own cells, which may be the reason for their low immunogenicity. This mini-review discusses the mechanisms of central and peripheral tolerance that may influence the formation of an anti-senescent immune response and be responsible for the accumulation of senescent cells with age.

## 1. Introduction

The development of medicine led to a significant increase in life expectancy in most countries [1]. According to the World Health Organization (WHO), the number of people over 60 will double by 2050 and reach 2.1 billion people [2]. The global trend toward an aging population leads to an increase in age-associated diseases and places a burden on the world economies [3,4,5]. However, as a result of scientific and technological progress, the arsenal of methods for treating age-related diseases is constantly expanding. In recent years, the possibility of using vaccines to prevent and treat the most common age-related diseases has been actively studied [6]. Thus, the first positive results were obtained from vaccines used to treat Alzheimer’s disease, type II diabetes mellitus, osteoarthritis, arterial hypertension, and some other cardiovascular diseases. Such vaccines can be based on different platforms and include peptide, protein, viral, cellular, and mRNA vaccines, as well as vaccines based on lipid particles (Table 1) [7].

A conceptually new approach is associated with the development of senolytic drugs. A number of studies have shown that aging of the body is accompanied by the accumulation of senescent cells in various tissues and organs [50,51]. This leads to tissue homeostasis and functional disorders typical of old age [52]. Senescent cells are cells with accumulated mutations, impaired autophagy, and defects in mitochondrial function, incapable of proliferating [53]. These cells are characterized by shortened telomeres, low heterochromatin orderliness, and defects in the nuclear membrane [54,55]. Morphologically, they are distinguished by an increased size and hypertrophy of the lysosomal apparatus [56,57,58]. Senescent cells also typically express some age-related markers (such as p16^INK4A^, p21^CIP1^, etc.), have increased senescence-associated β-galactosidase (SA-β-Gal) activity, produce a number of cytokines and pro-inflammatory substances (Senescence-Associated Secretory Phenotype—SASP), and have various defects in the protein quality control machinery [59,60,61,62,63]. The physiological features of senescent cells affect their antigen profiles [64]. Unique sets of mutations and abnormalities in post-translational modifications (PTMs) typical of senescent cells can alter the structure of various proteins [65,66]. In addition, the accumulating body of evidence points at the increased genomic instability, activation of retroelements, and awakening of some latent viruses in senescent cells, which also causes the emergence of new antigen determinants [67,68]. Thus, senescent cell antigens (SAs) can be divided into Senescent-Associated Antigens (SAAs) and Senescent-Specific Antigens (SSAs). The first case refers to unchanged self-antigens that have increased levels of expression on some types of senescent cells (e.g., uPAR) [69]. The second case refers to “neoantigens” that appeared de novo and are unique to senescent cells.

It is worth noting that senescent cells play an important role in some physiological processes. Thus, SASP factors are involved in tissue remodeling in early ontogenesis, as well as in repair and regeneration processes at later stages of development [70]. In addition, cell cycle arrest, which is the most important feature of senescence, prevents possible malignancy [71]. Senescent cells are normally present in tissues in limited numbers. However, the accumulation of these cells during the aging process is associated with the dysfunction of various tissues and organs, and the cumulative effect of SASP production contributes to chronic age-related inflammation, which increases the risk of developing age-related diseases [72]. At the same time, selective elimination of these cells during aging leads to the restoration of functions gradually lost with age and, in some cases, was demonstrated to extend the life of experimental animals [73,74].

Despite the promising results of using some drugs aimed at killing senescent cells (senolytics) or reducing the negative effects of SASP (senomorphics), the existing pharmacological approaches still do not possess the high specificity toward senescent cells, do not take into account their diversity, and are associated with the risk of side effects [75,76,77,78]. This significantly limits their translational potential. In this context, a promising alternative is the development of methods for targeted elimination of senescent cells with the help of adaptive immunity mechanisms. Various attempts to create senolytic vaccines that remove senescent cells from specific tissues have already been made. For example, vaccines against GPNMB (glycoprotein nonmetastatic melanoma protein B) and CD153 antigens showed positive results with minimal side effects [79,80]. In mice, targeting GPNMB with a peptide vaccine reduced senescence load, improved metabolic indices, and reduced atherosclerotic plaques [81]. Furthermore, the anti-CD153 peptide vaccine reduced the number of senescent T cells in adipose tissue, increased glucose tolerance, and improved the response to endogenous insulin [80]. In addition, CAR-T therapy against uPAR, a marker of senescent cells that can potentially be used in senolytic vaccines, showed a beneficial effect, increasing stamina and improving glucose tolerance in aged mice [69]. Targeting the senescent cell marker NKG2D with CAR-T selectively eliminates senescent cells in vitro with minimal impact on healthy cells, suggesting its therapeutic potential for senolytic vaccines [82]. However, the development of such vaccines is associated with certain problems. Unique SSAs absent in normal cells are still unknown, which hinders the development of safe senolytic vaccines due to the risk of damage to the healthy tissues [53,83]. The high antigenic diversity of senescent cells, both at individual and population levels, significantly complicates the search for target antigens for the creation of universal senolytic vaccines [53,64]. Obviously, tolerance to senescence-associated antigens is a serious problem [84,85]. Since senescent cells are the body’s own cells, the mechanisms of central and peripheral tolerance can act toward the antigens they express. They are carried out due to the elimination of autoreactive lymphocytes in the thymus and bone marrow, as well as through clonal inactivation and anergy of autoreactive clones in the periphery [86,87,88]. Thus, overcoming tolerance to senescence-associated antigens is one of the key challenges in the development of senolytic vaccines.

## 2. Central Tolerance for Senescent Antigens

Mechanisms of central tolerance are crucial for preventing autoimmune diseases. The removal of potentially autoreactive immature T and B lymphocytes occurs during negative selection in the thymus and bone marrow, respectively [89,90]. The key role in this process is played by the diversity of self-antigens that immature lymphocytes encounter during maturation [91]. Unlike the thymus, the bone marrow does not have a specialized system for presenting self-antigens. Therefore, lymphocyte selection is less strict and limited to the default set of self-antigens present in the bone marrow. At the same time, the aging of bone marrow cells can lead to the emergence of senescence-associated antigens and the formation of tolerance of B cells to these antigens [92,93].

The situation is different in the thymus, where a complex system of presentation of self-antigens exists [91]. The multitude of antigens presented in the thymus forms the clonal diversity of effector (Teff) and regulatory (Treg) T lymphocytes and ensures equilibrium in the immune system and its ability to respond to antigen challenges, maintaining protection against oncological, autoimmune, and infectious diseases [94,95]. Apparently, the thymus is simultaneously involved in the formation of an anti-senescent immune response and the elimination of senescent cells, as well as in maintaining tolerance to a multitude of SA [96,97,98]. The balance between these processes is determined by the repertoires of Teff and Treg cells that recognize SA [95]. In other words, the nature of the immune response to these antigens depends on their presentation in the thymus.

**Figure 1:**
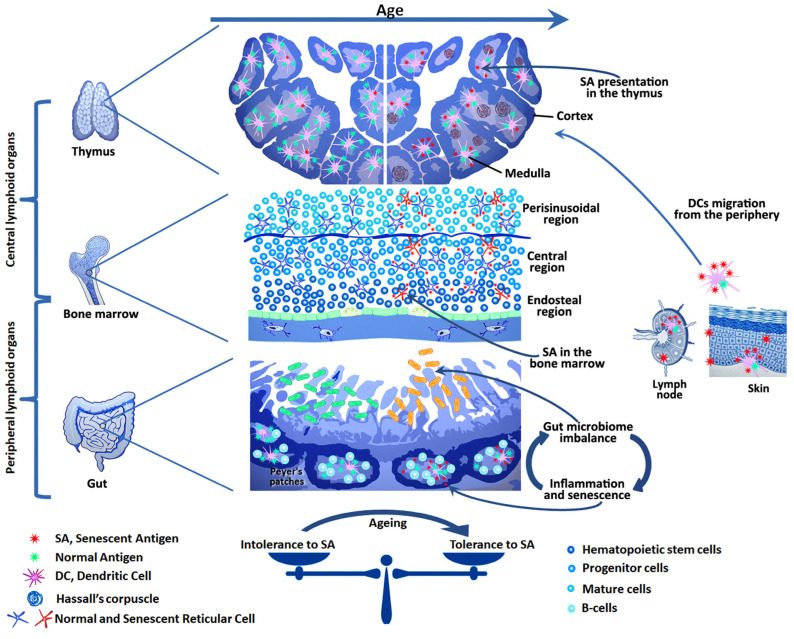
In youth, aged and senescent cells are efficiently eliminated by the immune system. However, with age, the effectiveness of this process declines. This may be due to both the aging of the immune system itself and the development of tolerance to senescent cell antigens. Chronic inflammation caused by SASP factors, as well as the ever-increasing antigenic load, may play a key role in this by leading to exhaustion, anergy, or trans-differentiation of anti-senescent effector cells to Tregs. Senolytic vaccines aim to restore the immune system’s ability to selectively eliminate senescent cells without compromising the organism as a whole. To achieve this, it is necessary to consider age-related changes in the system of autotolerance: the appearance of SA in the bone marrow, thymus, and periphery, as well as the microbiome imbalance, as these changes affect the repertoires of effector and regulatory T and B cells specific for SA.

The action of the transcription factors AIRE, FezF2, and DEAF1 in cooperation with the helicase CHD4 in the thymus provides promiscuous gene expression (PGE) of the overwhelming majority of tissue-specific antigens [91]. Along with alternative splicing, the diversity of self-antigens in the thymus increases due to the expression of endogenous retroelements [99]. In addition, some dendritic cells migrate to the thymus and carry peripheral antigens with them (see Figure 1). The intensity of this process increases with age, which may be the reason for the formation of central tolerance to some SA [100,101,102,103]. Thus, a unique library of central tolerance antigens is formed in the thymus, which ensures the selection of thymocytes and participates in the formation of T-cell repertoires [91]. However, this library undergoes qualitative and quantitative changes during life. The age-related thymus involution is accompanied by a decrease in the number of medullary thymic epithelial cells (mTECs), a decrease in PGE, an increase in the number of dendritic cells with peripheral antigens, and an accumulation of cells with signs of senescence in Hassall’s corpuscles [104,105,106]. This leads to a decrease in thymopoiesis (thymic output) and a decrease in the diversity of T-lymphocyte repertoires [107,108]. Apparently, age-related central tolerance to some SA is formed due to an increase in their share in the repertoire of antigens presented in the thymus. This is associated with the aging of the thymus itself and the appearance of senescent cells among mTEC and in Hassall’s corpuscles, as well as an increase in the influx of SA from the periphery due to the migration of dendritic cells [104,105,106]. Accurate identification and determination of the number of these antigens is a complex task. Nevertheless, the described mechanism is a probable cause of the low efficiency of promising senolytic vaccines aimed at SA.

The solution to the problem can be found in a more detailed examination of the aging processes in the thymus and periphery. As noted earlier, PTM processes are disrupted in aging and senescent cells [65,66,109]. This leads to changes in the immunopeptidome in these cells. On one hand, the appearance of a PTM peptide in the molecule of the major histocompatibility complex (MHC) can shield it from the T-cell receptor (TCR) [110]. On the other hand, such a peptide, on the contrary, is capable of causing a strong immune response [110]. The latter, for instance, can be observed in some autoimmune diseases, when a protein with the correct primary structure activates T cells due to the features of PTMs [111,112]. In the thymus, the intensity of PTMs is much lower than in the periphery, and PTM proteins are almost not involved in negative selection, which determines the absence of central tolerance towards many modified proteins [113]. Some authors consider this to be one of the main factors in the development of autoimmune diseases [111,112]. It is worth noting that due to the imbalance of pro- and anti-oxidant processes in senescent cells, non-enzymatic PTMs (such as cysteine redox PTMs, acyl transfers, glycation, formylation, etc.) are apparently most common, as are some enzymatic PTMs (such as mono- and poly-ADP-ribosylation) [65,109]. Thus, the appearance of specific modifications may be more likely in the senescent state and may not occur in normal cells. Therefore, further search for common patterns of PTMs characteristic of senescent cells, as well as identification of specific PTMs, may open prospects for the development of a relatively universal senolytic vaccine.

Another approach is related to the detection of the activity of transposable elements (TEs), DNA segments that are capable of moving within the genome and are genetic parasites [114]. This heterogeneous group includes more than 800 subfamilies and can be divided into the following three categories: LTR (Long Terminal Repeats), LINE, and SINE (Long and Short Interspersed Nuclear Elements, respectively). In total, TEs occupy approximately 45% of the human and mouse genomes [115]. Due to their mutagenic potential, TE activity is constantly suppressed in healthy cells. However, with age, their activity increases significantly and accompanies the transition of cells to a senescent state [116,117]. Interestingly, TEs play a physiological role in the thymus; their activity is increased in mTECs and in plasmacytoid dendritic cells, where TEs enrich MHC-I immunopeptide, thus participating in the negative selection of thymocytes [115]. Despite this, many TE protein products are highly immunogenic, which is associated with an increase in the risk of autoimmune diseases with age due to the activation of TEs in various tissues [117,118,119,120]. This suggests the formation of incomplete tolerance to the entire diversity of TE antigens. Currently, there is very limited data on the activity of specific TE subfamilies in the thymus, depending on age. In general, the highest activity of LINE and SINE is observed at a young age and decreases with thymus involution [115]. No such general pattern of decreased activity is observed for LTR subfamilies. Some of them are inactivated in early ontogenesis, while others demonstrate high activity in mTECs both before and after thymus involution [113]. Aging is generally associated with the activation of all TE categories. However, a common pattern characteristic of senescent cells is an increase in the activity of some LINE1 subfamilies and even the formation of retrovirus-like particles [121]. Thus, the search for the most characteristic senescent cell subfamilies of TEs with low activity in the thymus, as well as the identification of their protein products, opens up opportunities for the creation of effective senolytic vaccines directed against various types of senescent cells.

In addition, a recent study demonstrated that during skin aging, latent cytomegalovirus is awakened in senescent fibroblasts. This leads to the presentation of glycoprotein B epitopes in MHC-II molecules and the destruction of senescent cells by CD4^+^ lymphocytes with cytotoxic action [122,123]. It is plausible to speculate that cell aging is associated with the activation of other dormant pathogenic or commensal viruses, and the intensification of the immune response against them can serve to eliminate senescent cells [124,125]. Therefore, a detailed study of the genetic and antigen profiles of senescent cells, taking into account the features of the formation of central tolerance, opens up broad prospects for the development of universal, safe, and effective senolytic vaccines.

## 3. Peripheral Tolerance for Senescent Antigens

Negative selection in the thymus is not absolutely efficient, and a significant portion of potentially autoreactive T cells end up in the periphery. This requires the presence of peripheral mechanisms that suppress autoreactive cells and ensure irresponsiveness to self-antigens. The mechanisms of peripheral tolerance are very diverse and are well described by now [126,127]. In general, they are aimed at preventing or limiting the activation of autoreactive T and B cells. The tolerance of autoreactive lymphocytes to cognate antigens is achieved through various mechanisms: quiescence, ignorance, anergy, exhaustion, deletion of clones via the induction of apoptosis, and finally senescence [128]. It is worth noting that under conditions favorable for the induction of tolerance, lymphocytes of any specificity can be inactivated through these mechanisms. This can lead to the formation of tolerance to antigens that the immune system previously perceived as foreign [128]. Activation of a naive B cell is impossible without a T lymphocyte of the appropriate specificity, in particular due to the follicular exclusion mechanism [86,129]; therefore, further description of peripheral tolerance will be given from the T lymphocyte perspective. Naive T cells emerging from the thymus recognize various self-antigens in the peripheral area with low specificity. This provides them with a tonic TCR signal necessary for survival and homeostasis. At the same time, the intensity of this signal is below the activation threshold, which keeps lymphocytes in a quiescent state [130]. The mechanisms of ignorance are poorly understood; apparently, they are associated not only with low TCR specificity but also with low density and availability of self-antigens for recognition by naive T cells [86]. Deficiency of co-stimulation underlies T cell anergy and prevents activation of naive autoreactive T lymphocytes upon interaction with antigen-presenting cells (APCs) [131]. Exhaustion is typical of Teff cells that receive antigenic stimulation for a long time, for example, during persistent infections or autoimmune inflammation [132]. Exhausted cells react weakly to cognate antigens and produce cytokines poorly compared to normal Teff cells. These cells express inhibitory molecules (such as PD1, LAG3, TIGIT, CD38, CD39, TIM3, etc.) and also have special epigenetic, transcriptional, and metabolic features that do not allow them to enter a quiescent-like cell state, characteristic of memory T cells (Tmem) [86,132]. Like other cells, lymphocytes are also subject to aging. Repeated antigenic stimulation of TCR can lead to replicative exhaustion, shortening of telomeres, decreased reactivity, and other signs of senescence. Although lymphocyte senescence is poorly understood, there is evidence of both low reactivity and proliferative potential of these cells [133,134,135]. One of the most important mechanisms of peripheral tolerance is the deletion of autoreactive cells [136,137,138]. Death by apoptosis can occur in different situations. To form an immune response, activation of a threshold number of T cells or otherwise achieving a quorum of T cells is necessary [139]. Without the necessary quorum, the immune response does not develop, and previously activated T cells are likely to die by apoptosis [140]. Also, deletion of lymphocytes through apoptosis occurs with insufficient stimulation, and only a small part of these cells goes into anergy. Interaction with tolerogenic APCs or Tregs usually ends in the death of the lymphocyte. However, occasionally it leads to the induction of peripheral Tregs [141]. This is an essential mechanism of peripheral tolerance, which plays an important role in expanding the spectrum of antigens to which relatively stable tolerance is formed in the periphery.

Age-related changes in the repertoires of peripherally presented antigens can lead to activation or, conversely, suppression of the immune response due to the mechanisms described above [139,142]. Apparently, depletion and transition of T lymphocytes to a senescent state are important factors in suppression of the immune response during prolonged persistence of antigens. This is observed in various chronic infections or malignant processes [139]. At the same time, the relationship between persistent infections and the development of autoimmune diseases is well known, which implies non-specific activation of adaptive immunity and violation of auto-tolerance [143,144]. This relationship is largely explained by genetic factors, dysfunction of Treg cells, and antigen mimicry. However, there is another important mechanism associated with an increase in the probability of achieving a quorum by potentially autoreactive T cells. Thus, during acute or short-term infections, high-amplitude activation of a small number of T-lymphocyte clones specific to the most immunogenic antigens occurs [139,145]. In long-term persistent infections, the diversity of antigens that activate different clones of T cells is significantly higher. This is due to the duration of the process, tissue damage, and an increased availability of some autoantigens [139,146]. All this leads to an expansion of the repertoire of T lymphocytes involved in the immune response and increases the likelihood of the involvement of cross-reactive T cells capable of recognizing self-antigens. Moreover, the activation of such cells and their transition from naive cells to Teff and Tmem cells is accompanied by a decrease in the activation threshold due to increased expression of co-stimulation receptors, as well as oligomerization and formation of TCR nanoclusters [147,148]. A decrease in the activation threshold as well as a significant increase in the probability of achieving quorum by potentially autoreactive cells under inflammatory conditions are critical risk factors for the breakdown of auto-tolerance in persistent infections and oncological processes.

Senescent cells are constantly formed throughout life. With age, they accumulate in tissues. The constant expansion of the SA spectrum leads to an increase in the clonal diversity of anti-senescent T lymphocytes. Together with the production of inflammatory SASP factors, this increases the probability of activation of the threshold number of potentially autoreactive T lymphocytes [149]. This, apparently, is one of the risk factors for the development of autoimmune reactions associated with aging [150]. At the same time, senescent cells have developed certain mechanisms for evading immune surveillance. They increase the expression of HLA-E (H2-Qa-1), which protects them from NK and CD8^+^ lymphocytes, and also express the molecules CD24, CD47, and GD2, which transmit the “do not eat me” signal [85,151]. Apparently, the age-related decrease in the efficiency of elimination of senescent cells by the immune system is associated with the processes described earlier for persistent antigens. A gradual increase in the antigen load exhausts the reserves of the immune system; constant stimulation leads to the depletion of specific T cells or their transition to a senescent state.

Age-related changes in the repertoires of antigen-recognizing receptors of T and B lymphocytes are directly related to the dynamics of the diversity of commensal antigens. There is an increasing body of evidence pointing at the influence of the intestinal microbiome on the aging processes associated with the accumulation of senescent cells, an increase in systemic inflammation, and an increased predisposition to various age-related diseases [152]. Recent studies demonstrated that constant stimulation of the intestinal microbiome with antigens causes aging of B cells of the germinal centers in the lymph nodes of the small intestine. This leads to the accumulation of B cells with signs of senescence and a shift in the diversity of B cell-produced IgA molecules. In turn, this disrupts the balance of the microbiome and causes additional antigen stimulation, which closes the vicious circle (see Figure 1) [153]. Chronic local inflammation leads to the damage of tissue barriers and promotes bacterial translocation (the penetration of bacteria or their antigens into the systemic bloodstream) [154,155]. Chronic inflammation and stimulation with multiple commensal antigens lead to imbalance in the repertoires of antigen-recognizing receptors of Teff and Treg cells, accumulation of Tmem cells, and a decrease in the number of naive lymphocytes [156,157,158]. Under these conditions, the activation threshold of a large number of T cells of various specificities decreases and the probability of achieving quorum by autoreactive lymphocytes increases. In addition, it has been shown that disturbances in the intestinal microbiome cause a decrease in the efficiency of hematopoiesis due to the suppression of multipotent progenitor cells [159]. Thus, constant antigen stimulation depletes the immune system and is one of the causes of immunoaging and decreased immunoreactivity in old age. This is reflected in an increased risk of autoimmune and oncological processes, as well as an increased susceptibility to infectious diseases and reduced vaccination efficiency in the elderly [160]. Therefore, the development of senotherapy methods aimed at reducing the number of senescent cells in the gastrointestinal tract along with restoring microbiome balance may be a promising strategy to mitigate the negative consequences of aging. In this context, the development of senolytic vaccines aimed at eliminating senescent B lymphocytes in the germinal centers of the small intestinal lymph nodes represents an example of a potential tissue-specific vaccination strategy. This is of interest from the point of view of the uniqueness of the pathogenetic mechanisms of aging inherent in each tissue or organ. Age-related changes in the microbiome adversely affect the bone marrow output of B cells, their differentiation, and antibody production [161]. The spontaneous development of germinal centers observed with aging, coupled with the accumulation of senescence-associated T and B cells, contributes to immunoaging and increases the risk of autoimmune reactions [162]. These changes, in turn, diminish the effectiveness of vaccination and elevate the risks of adverse reactions. This underscores the necessity for further research into the role of the microbiome in shaping the immune response to vaccination, as well as the development of effective strategies for microbiome correction in older individuals, particularly during vaccination periods. Recent studies have demonstrated that bifidobacterium can significantly enhance the vaccine response and represent an important component of the microbiome in youth [163]. It is evident that vaccination, when combined with microbiome correction, may enhance the efficacy and safety of the use of senolytic vaccines in older individuals.

Thus, the development of autoimmune reactions appears to be the greatest threat to the intensification of the immune response against SA, and both the formed tolerance to SA and the reduced immune reactivity typical of old age create additional difficulties in the development of senolytic vaccines [164]. Despite the attractiveness of such platforms and the demand for vaccine-based approaches for the prevention and therapy of age-related diseases, the development of such methods still presents a serious challenge for modern immunology and biotechnology [165]. However, alternative methods of combating the pathological accumulation of senescent cells, which rely on various senolytics and senomorphics, are currently being widely studied [166,167]. Perhaps the use of approaches combining specific stimulation of the immune response with the use of other methods of senotherapy will provide a safe and effective method for reducing the senescent load.

## 4. The Current State of the Senotherapeutic Approaches

In the last decade, encouraging results have been obtained in the study of senolytics, drugs that selectively destroy senescent cells by inducing apoptosis [168]. The demonstrated positive effect of dasatinib and quercetin on senescent cells led to widespread research into senolytics [169]. These drugs are able to induce apoptosis in senescent cells by suppressing ephrins (EFNB-1/3) and phosphatidylinositol 3-kinase (PI3K), respectively [169]. These targets play an important role in the molecular cascades that control senescent cell survival and apoptotic death, and their blockade causes the elimination of senescent cells of various localizations [169,170]. Although the precise mechanisms of action of senolytics are not yet fully understood, it is hypothesized that transcriptomic differences between healthy and senescent cells confer a degree of selectivity in the effects of these drugs [67,171]. The need to increase efficiency and specificity while reducing the risk of off-target effects led to the emergence of the second generation of senolytics. The substances in this group are diverse and can act through a wide range of senolytic mechanisms. These include inhibition of intracellular pathways essential for senescent cells, depletion of metabolites critical for these cells, disruption of proteostasis, electrolyte balance, aggravation of mitochondrial dysfunction, promotion of ROS accumulation, and many others. [170]. The main molecular targets of the second generation of senolytics include proteins of the Bcl-2 family, p16, p21, p53, Akt, HSP90, PI3K, FOXO4, SA-β-Gal, and others [170,172]. In addition, the action of some substances from this group, such as cardiac glycosides, is associated with suppression of Na^+^/K^+^-ATPase, which leads to destabilization of the membrane potential and a decrease in intracellular pH [173]. It is assumed that senescent cells are already at the limit of their adaptive reserve, and additional stress leads to their selective death.

Another strategy of senotherapy is aimed at minimizing the impact of senescent cells on the body. Usually, drugs from this group (senomorphics) inhibit the production of pro-inflammatory SASP factors in various ways without killing senescent cells [167]. The most well-known representatives of senomorphics are rapamycin, metformin, quercetin, resveratrol, aspirin, and statins [174]. The mechanisms of action of this group of substances are very diverse and have not been sufficiently studied. Most often, their action is directed at the molecular targets of the following signaling pathways: mTOR, NF-κB, AMPK, SIRT1, IGF-1, NRF2, p38MAPK, IRAK1, etc. [153,174]. Interestingly, more and more data are accumulating that senescent cells can perform useful physiological functions [175]. For example, it was demonstrated that in some cases, their elimination can negatively affect the structure of tissues, slow down regeneration and wound healing, and, at the early stages of development, even lead to disruption of the development of organs and tissues [175,176]. Therefore, in some situations, the use of senomorphics may be more preferable. The uniqueness of the aging mechanisms for each cell type is associated with the functional and structural features of various tissues and organs. Several SAAs have been identified. Some are common to various senescent cell types (e.g., NKG2DL and uPAR), while others exhibit tissue specificity (e.g., Cathepsin F in dermal fibroblasts, CD9 in endothelial cells, KLRG-1 and CD153 in T cells, and CD30 in B cells; see Table 2) [69,80,82,177,178]. This suggests the potential for tissue-specific elimination of senescent cells, which is important in the context of the uneven aging of different tissues [179]. However, to minimize off-target effects, further investigation into both common and tissue-specific SSAs is required. This would broaden the therapeutic potential of targeted senotherapeutic approaches, including vaccine-based strategies.

The diversity of the nature of senescent cells explains the selectivity of the action of modern senotherapeutics in relation to certain cell types or subpopulations of senescent cells. Therefore, combinations of some senotherapeutic agents that demonstrated synergistic action and efficiency are currently being studied [169,170,172]. The most studied senotherapeutics include various combinations of dasatinib, quercetin, fisetin, and navitoclax, which have been tested in clinical trials and shown positive results [166,189]. It should be emphasized that the causes and mechanisms of cellular aging differ for different physiological or pathological processes. This implies the uniqueness of the signs and markers of aging and senescent cells inherent in a specific cell type, tissue, or organ. Therefore, the development of senotherapeutic strategies should take into account not only the fundamental principles of aging but also the specific pathophysiological characteristics of the organism as well as the target tissue or organ. Therefore, the development of senotherapeutic strategies should take into account the specific pathophysiological characteristics of the organism as well as the target tissue or organ. Also promising are strategies targeting the fundamental causes of senescent cell accumulation. For example, selective activation of apoptosis in senescent cells while sparing healthy cells would allow the removal of harmful and dysfunctional cells with minimal side effects. Thus, a primary goal of senotherapy is the safe mitigation of aging’s effects and the prolongation of a healthy lifespan.

## 5. Discussion

The use of various senotherapy strategies opens up great opportunities for extending the period of active longevity. Positive effects from the use of various senolytics and senomorphics have been demonstrated in recent years [190,191]. However, their action is still insufficiently specific, is associated with the risk of adverse reactions, and requires higher efficiency [192]. Relatively high specificity and efficiency in experiments on mice were demonstrated by removing senescent cells using cytotoxic antibodies or CAR-T therapy [69,180,193,194,195]. However, in the context of widespread use, these approaches are significantly inferior to senolytic vaccines due to their extremely high cost, difficulty of production, and transportation.

Vaccine development is, in principle, a complex and lengthy process that includes searching for and selecting a target antigen, choosing a platform and adjuvant, and assessing efficacy and safety. The latter is the most important point before widespread clinical use is approved. The development of senolytic vaccines opens a fundamentally new stage in the development of vaccine technology, which will require overcoming many pitfalls and hidden risks, primarily those related to safety. It is currently assumed that the restoration of the functions of old tissues and organs will be associated with the removal and replacement of senescent cells with new intact cells with normal vital activity. However, how timely and effective this process will be in various aged tissues remains unknown. For example, the depletion of cells positive for the senescence marker p16 in mouse models caused damage to the hemato-tissue barriers and led to fibrosis in various organs, while the removal of senescent endothelial cells did not lead to their replacement with new cells [196]. This demonstrates the possible negative effects of the elimination of senescent cells in various tissues, which must be taken into account when developing senolytic methods. Mass removal of senescent cells after vaccination can be associated with the development of a dangerous inflammatory response or the triggering of autoimmune reactions [197,198,199,200]. This must be taken into account in the context of the development of prophylactic or therapeutic vaccines. Apparently, preventing the accumulation of a large number of senescent cells in tissues by preventive vaccination carries fewer risks than using a therapeutic approach. In order to minimize the associated risks, senotherapeutic vaccines may be aimed at a narrower range of targets, act on specific target tissues or organs, and take into account their regenerative potential. Hypothetically, senolytic vaccines could be utilized for both therapeutic and preventive purposes. In the therapeutic context, it is proposed that they would eliminate accumulated senescent cells and restore tissue functions. In the preventive context, these vaccines may be administered at a younger age to prevent the age-related accumulation of senescent cells and reduce the likelihood of age-related changes. This is a promising prospect that necessitates further investigation.

Thus, in addition to identifying strictly specific antigens and obtaining an effective immune response that eliminates senescent cells, it is necessary to assess the potential risks associated with the removal of these cells. It is necessary to consider how the elimination of senescent cells will affect the function of the tissue or organ, as well as whether the regenerative potential is sufficient enough to replenish the lost elements in a timely manner [201]. It is impossible to “cancel” the formed immune response to the vaccine, so minimizing the likelihood of developing an excessive inflammatory response and triggering autoimmune processes is a critical aspect of the development of senolytic vaccines. Obviously, the creation of senolytic vaccines for various purposes will allow the use of personalized approaches for the prevention or treatment of certain age-associated diseases, as well as for the implementation of other anti-aging strategies.

## 6. Conclusions

In the last 10 years, the rapid development of genetic and omics technologies has opened up great opportunities for detailed research of various aspects of cell aging. High-resolution proteome assessment and single-cell transcriptome studies facilitate the search for new senescence-associated and senescence-specific antigens and allow for a more in-depth analysis of the heterogeneity of senescent cells within a tissue, organ, or between individuals. The novel coronavirus pandemic has accelerated the advancement of various platforms for the development of highly effective and safe vaccines. The new approaches rely on various adjuvants to selectively stimulate humoral or cellular immunity [202,203,204]. In vitro and in silico models are being developed for personalized prediction of adverse effects from vaccination [205,206,207]. In the long term, this will help overcome the main difficulties associated with the search for specific antigens absent in healthy tissues and develop a vaccine-based approach for safe and effective stimulation of the immune response to eliminate senescent cells of a specific type or a more universal method. Therefore, continued research in the field of senotherapy and the development of senolytic vaccines has great potential and opens up prospects for extending the period of active and healthy longevity.

## Figures and Tables

**Table 1:** Vaccine approaches for combating age-related diseases.

Disease	Target	Vaccine Design	Stage
Alzheimer’s disease	Amyloid beta (Aβ)	Palmitoylated peptide Aβ1–15 (for ACI-24) was reconstituted in liposomes.	Phase II clinical trials (NCT05462106) [8,9,10]
		UB-311 comprises two Aβ1–14–targeting peptides (B-cell epitope), each synthetically linked to different helper T-cell peptide epitopes (UBITh^®^), and formulated in an alum-containing Th2-biased delivery system.	Phase II clinical trials (NCT00965588; NCT02551809; NCT03531710) [11,12]
		AV-1959 consists of pVAX1 backbone vector which codes a protein consisting of the Ig κ- chain signal sequence, three copies of the A1-11 B cell epitope, one synthetic peptide (PADRE), and a string of non-self-promiscuous Th epitopes from Tetanus Toxin, hepatitis B virus and influenza.	Phase I clinical trials (NCT05642429) [13]
		Aβ33–40 peptide coupled to monomeric keyhole limpet hemocyanin suspended in the phosphate buffer with 0.35% aluminum hydroxide.	Phase II clinical trials (NCT03113812; NCT03461276) [14]
	Microtubule-associated protein tau (Tau)	ACI-35 is a liposomal vaccine that carried peptide corresponding to residues 393–408 in protein Tau (numbering of the Tau441 isoform) with S396 and S404 phosphorylated.	Phase II clinical trials (NCT04445831) [15]
		Axon Peptide 108 (N-terminally cysteinylated tau 294–305/4R) coupled to keyhole limpet hemocyanin via a maleimide linker, with aluminum hydroxide adjuvant.	Phase II clinical trials (NCT01850238; NCT02031198; NCT02579252) [16,17,18]
	Mimicking the extra-telomeric human telomerase reverse transcriptase (hTERT)	GV1001 is a 16-amino-acid vaccine peptide derived from the human telomerase reverse transcriptase (hTERT) sequence.	Phase II clinical trials (NCT05189210) [19]
	Proteasomes	Nasal vaccination with IVX-908 (Protollin) which is a non-covalent formulation of outer membrane proteins (proteasomes) of *Neisseria meningitides* and lipopolysaccharide from *Shigella flexneri* plus glatiramer acetate (GA).	Preclinical studies, mice [20]
Type 2 diabetes	Dipeptidyl peptidase-4 (DPP4)	Peptides E1 and E2, which spans a site in the N-terminal sequence of DPP4; E3, which spans the 89–97 a.a. sequence near the opening of the DPP4 active site were conjugated to keyhole limpet hemocyanin.	Preclinical studies, mice [21]
	Interleukin-1 beta (IL-1β)	A rhesus (rmIL1bQb) and human (hIL1bQb) version of the IL-1β vaccine were generated with an identical inactivating mutation of the IL-1β polypeptide. The corresponding rhesus monkey (rmIL1bQb) and human (hIL1bQb) IL-1β vaccines were produced by chemically crosslinking the engineered IL-1β proteins to Qb virus-like particles.	Phase I clinical trials (NCT00924105) [22]
		IL-1β epitope peptide formulated with alum-based adjuvant.	Preclinical studies, mice [23]
	Prorenin	Three different epitopes of the prorenin prosegment (E1, E2, and E3) were selected and conjugated to keyhole limpet hemocyanin.	Preclinical studies, mice [24]
Hypertension	Angiotensin I	PMD-2850 contains tetanus toxoid, and PMD-3117 contains keyhole limpet hemocyanin; both are conjugated to Angiotensin I peptide analog and use aluminum hydroxide gel as adjuvant	Phase I clinical trials [25,26]
	Angiotensin II	An angiotensin II-derived peptide was conjugated to the virus-like particles Qbeta (AngQb).	Phase II clinical trials (NCT00500786; NCT00701649; NCT00710372) [27,28]
	Angiotensin 1 receptor (AT1R)	Qβ virus-like particle protein was covalently conjugated to ATR-001 peptide with the amino acids sequence corresponding to the sequence of the second extracellular loop of the human AT1R.	Preclinical studies, mice [29,30]
	Apha-1D adrenergic receptor (ADRA1D)	Epitopes of α1A-adrenergic receptor (α1A-AR) or α1D-adrenergic receptor (α1D-AR) were conjugated to Qβ bacteriophage virus-like particle.	Preclinical studies, mice [31]
Abdominal aortic aneurysm	Angiotensin II	Angiotensin II peptide was conjugated to keyhole limpet hemocyanin.	Preclinical studies, rats [32]
	Angiotensin type 1 receptor (AT1R)	Qβ virus-like particle protein was covalently conjugated to ATR-001 peptide with the amino acids sequence corresponding to the sequence of the second extracellular loop of the human AT1R position 180–187.	Preclinical studies, mice [33]
Atherosclerosis	Apolipoprotein B (ApoB)	A vaccine composed of ApoB peptide and complete Freund’s adjuvant.	Preclinical studies, mice [34]
	Proprotein convertase subtilisin/kexin type 9 (PCSK9)	A short PCSK9 peptide (as a B cell epitope) is linked to a tetanus peptide (as a T cell epitope). The peptide was conjugated to the surface of nanoliposome carriers and formulated with alum vaccine adjuvant.	Preclinical studies, mice [35]
	A disintegrin and metalloproteinase with thrombospondin type 1 motif 7 (ADAMTS7)	Three potential vaccines consist of distinct B cell epitope peptides derived from ADAMTS-7 and conjugated with the carrier protein keyhole limpet hemocyanin as well as aluminum hydroxide as an adjuvant.	Preclinical studies, mice [36]
	Lysate-based	Contain pooled antigens derived from pig adipose tissue.	Phase III clinical trials (NCT03042741) [37,38]
Osteoarthritis	Nerve growth factor (NGF)	Virus-like particles were derived from the cucumber mosaic virus (CuMV) and coupled to expressed recombinant NGF to create the vaccine.	Preclinical studies, mice [39]
Fibrosis	Disintegrin and metalloproteinase domain-containing protein 12 (ADAM12) and Glioma-associated oncogene family zinc finger 1 (GLI1)	Lentiviral vectors with ADAM12 or GLI1 full-length protein coding sequence and incomplete Freund’s adjuvant plus the TLR9 agonist CpG oligodeoxynucleotides.	Preclinical studies, mice [40]
	Proprotein convertase subtilisin/kexin type 9 (PCSK9)	Epitope peptide of PCSK9-003 was derived from human PCSK9 and conjugated to Qβ virus-like particles.	Preclinical studies, mice [41]
Parkinson’s Disease and multiple system atrophy	Alpha-synuclein	AFFITOPE^®^ PD01A and PD03A are peptides mimicking an epitope in the C-terminal region of human alpha-synuclein but with a different amino acid sequence. Every protein is conjugated to the carrier protein keyhole limpet hemocyanin and adsorbed to aluminum hydroxide adjuvant.	Phase II clinical trials (NCT02618941; NCT02267434; NCT02270489) [42]
		UB-312 a 10-amino-acid fragment from the alpha-synuclein C-terminus is fused to a small peptide UBITh^®^ that activates T-helper cells, and combined with adjuvant composed of polyanionic Cytosine phosphoguanine (CpG), oligodeoxynucleotide (ODN), and aluminum-based adjuvant (Adju-Phos^®^).	Phase II clinical trials (NCT04075318; NCT05634876) [43,44]
Neovascular maculopathy and age-related macular degeneration	Vascular endothelial growth factor receptor 1 and 2 (VEGFR1 and 2)	An HLA-A*2402 or A*0201 restricted epitope peptides of VEGFR1 and VEGFR2 emulsified with Montanide ISA 51.	Clinical trials 1 phase (NCT00791570)
Obesity	Somatostatin	A chimeric-somatostatin with either JH17 or JH18 adjuvants.	Preclinical studies, mice [45]
	Glucose-dependent insulinotropic polypeptide	Immunoconjugate of glucose-dependent insulinotropic polypeptide covalently attached to the Qβ bacteriophage virus-like particles.	Preclinical studies, mice [46]
	Ghrelin	A common porcine–rat–human ghrelin sequence is conjugated to BSA and emulsified in Freund’s incomplete adjuvant and diethylaminoethyldextran.	Preclinical studies, pigs [47]
		Synthetic ghrelin residues 1–10 for Ghr1, 13–28 for Ghr2, and 1–28 for Ghr3, coupled to the carrier protein keyhole limpet hemocyanin.	Preclinical studies, rats [48]
		Chemical conjugation of ghrelin with NS1 protein tubules from the Bluetongue Virus (BTV) as a carrier.	Preclinical studies, mice [49]

**Table 2:** Promising antigens for senolytic vaccine development.

Target	Cell Type	Organism	Description
ApoD (Apolipoprotein D)	Senescent skin fibroblast	Human	ApoD expression was upregulated in cellular senescence models and correlated with senescence-associated β-galactosidase activity and decreased proliferation, which was concomitant with the upregulation of SASP genes. ApoD-positive cells were found to be more abundant in the aging human dermis, which makes ApoD a promising target for senolytic vaccines [180,181].
CD153 (Tumor Necrosis Factor Ligand Superfamily Member 8)	Senescence-associated T cells in adipose tissue and spontaneous germinal centers	Mice	Anti-CD153 peptide vaccine reduced the number of senescent T cells in adipose tissue, increased glucose tolerance, and improved the response to endogenous insulin [80,177].
CD30 (Tumor Necrosis Factor Receptor Superfamily Member 8)	Senescence-associated T and B cells in spontaneous germinal centers	Mice	Blockade of the CD153/CD30 interaction via an anti-CD153 antibody suppresses the immune senescence phenotype, lupus, and ameliorates inflammaging [177].
CD87 (Urokinase Plasminogen Activator Surface Receptor)	Wide range of senescent cells	Mice	uPAR-positive senescent cells can be safely targeted with senolytic CAR-T cells. Such treatment improves exercise capacity in physiological aging, and it ameliorates metabolic dysfunction in aged mice and in mice on a high-fat diet [182].
CD9 (Tetraspanin-29)	Senescent endothelial cells	Mice, Human	CD9 is upregulated in senescent endothelial cells, neointimal hyperplasia, and atherosclerotic plaques. CD9 may be a novel target for the prevention and treatment of vascular aging and atherosclerosis. Therefore, CD9 could potentially be used as a target in senolytic vaccines [183].
CTSF (Cathepsin F)	Senescent skin fibroblasts and keratinocytes	Human	Cathepsin F is associated with the senescence of human fibroblasts and keratinocytes, making it a promising target for senolytic vaccines [178].
EGF (Epidermal Growth Factor)	Umbilical vein endothelial cells, lung fibroblasts	Human	EGF treatment can induce cellular senescence via the activation of the EGF receptor. Inhibition of EGF represents a promising treatment against cellular senescence and could be used as a target for senolytic vaccines [184].
GPNMB (Glycoprotein Nonmetastatic Melanoma Protein B)	Senescent endothelial cells, adipocytes, and leukocytes	Mice	The authors identify GPNMB as a senescence-associated antigen and a promising molecular target for senolytic therapy. Immunization of mice against GPNMB improved metabolic parameters, glucose tolerance, and reduced atherosclerotic plaques [81,185,186].
KLRG-1 (killer-cell lectin like receptor G1)	Senescent T cells	Mice, Human	KLRG1 is expressed on NK cells and antigen-experienced T cells and has been postulated to be a marker of senescence. The elimination of KLRG1-positive cells results in lasting rejuvenation of the immune system [187,188].
NKG2DL (Activating Receptor Natural Killer Group 2, Member D Ligand)	Senescent models of mouse embryonic fibroblasts and astrocytes	Mice	The authors demonstrate elevated expression of NKG2DLs in response to genotoxic and oxidative stress in senescent models. NKG2D CAR-T cells displayed potent and selective cytotoxicity against these senescent cells, suggesting their potential as targeted senolytics [82].
